# ST6GAL1 sialyltransferase promotes acinar cell survival and tissue regeneration during pancreatitis

**DOI:** 10.64898/2026.09.26.754701

**Published:** 2026-09-28

**Authors:** Michael P. Marciel, Nikita Bhalerao, Barnita Haldar, Sejal S. Shinde, Joshua C. Anderson, Christopher D. Willey, Srikanth Iyer, Vikas Dudeja, Susan L. Bellis

## Abstract

The role of aberrant glycosylation in pancreatitis remains an under-investigated area of research. Here, we determined that the ST6GAL1 sialyltransferase, which adds α2-6-linked sialic acids to *N* -glycoproteins, was upregulated in pancreatic tissues from patients with acute (AP) and chronic (CP) pancreatitis, and in mice with experimental AP. Within these tissues, ST6GAL1 was selectively expressed in acinar cells undergoing acinar to ductal metaplasia (ADM), a process whereby injured acinar cells de-differentiate and re-enter the cell cycle to enable tissue repair. To study the functional role of ST6GAL1 in pancreatitis, we used HPNE pancreatic epithelial cells with modulated ST6GAL1 expression, along with pancreatic organoids from mice with transgenic expression of ST6GAL1 (“SC” mice). In these models, ST6GAL1 activity promoted the activation of EGFR (a well-known ADM-driver), ERK, and AKT. ST6GAL1-expressing cells also displayed an ERK-dependent upregulation of the anti-apoptotic proteins, Mcl-1 and Bcl-xL, and impaired apoptotic signaling by the TNFR1 and Fas death receptors. Unbiased kinomics profiling revealed that ST6GAL1 induced the activation of many receptor tyrosine kinases associated with cell survival and proliferation (e.g., EGFR, PDGFR, MET). Furthermore, ST6GAL1 enhanced the survival and proliferation of cells exposed to stress-inducing conditions such as serum withdrawal. Based on these findings, we hypothesized that the pro-survival phenotype imparted by ST6GAL1 would facilitate tissue healing following a bout of pancreatitis. Accordingly, we induced AP in wild type and SC mice via L-arginine injection and found that SC mice had more rapid and efficient tissue repair, evidenced by accelerated restoration of the acini, diminished acinar apoptosis, and reduced immune cell infiltration. Together, these findings highlight a novel glycosylation-dependent mechanism that drives cell survival, positioning ST6GAL1 as a key adaptive mediator of pancreatic regeneration.

## Full Text Availability

The license terms selected by the author(s) for this preprint version do not permit archiving in PMC. The full text is available from the preprint server.

